# Association of left atrial strain by cardiovascular magnetic resonance with recurrence of atrial fibrillation following catheter ablation

**DOI:** 10.1186/s12968-021-00831-3

**Published:** 2022-01-03

**Authors:** Mina M. Benjamin, Naeem Moulki, Aneeq Waqar, Harish Ravipati, Nancy Schoenecker, David Wilber, Menhel Kinno, Mark Rabbat, Thriveni Sanagala, Mushabbar A. Syed

**Affiliations:** 1 Division of Cardiovascular Medicine, Loyola University Medical Center, Stritch School of Medicine, Maywood, IL USA; 2 Department of Internal Medicine, Loyola University Medical Center, Maywood, IL USA; 3 Department of Internal Medicine, MacNeal Hospital, Berwyn, IL USA

**Keywords:** Atrial fibrillation, Left atrial strain, Cardiovascular magnetic resonance, Catheter ablation, Recurrence

## Abstract

**Background:**

Atrial fibrillation (AF) is a progressive condition, which is characterized by inflammation/fibrosis of left atrial (LA) wall, an increase in the LA size/volumes, and decrease in LA function. We sought to investigate the relationship of anatomical and functional parameters obtained by cardiovascular magnetic resonance (CMR), with AF recurrence in paroxysmal AF (pAF) patients after catheter ablation.

**Methods:**

We studied 80 consecutive pAF patients referred for ablation, between January 2014 and December 2019, who underwent pre- and post-ablation CMR while in sinus rhythm. LA volumes were measured using the area–length method and included maximum, minimum, and pre-atrial-contraction volumes. CMR-derived LA reservoir strain (ℇR), conduit strain (ℇCD), and contractile strain (ℇCT) were measured by computer assisted manual planimetry. We used a multivariate logistical regression to estimate the independent predictors of AF recurrence after ablation.

**Results:**

Mean age was 58.6 ± 9.4 years, 75% men, mean CHA_2_DS_2_-VASc score was 1.7, 36% had prior cardioversion and 51% were taking antiarrhythmic drugs. Patients were followed for a median of 4 years (Q1–Q3 = 2.5–6.2 years). Of the 80 patients, 21 (26.3%) patients had AF recurrence after ablation. There were no significant differences between AF recurrence vs. no recurrence groups in age, gender, CHA_2_DS_2_-VASc score, or baseline comorbidities. At baseline, patients with AF recurrence compared to without recurrence had lower LV end systolic volume index (32 ± 7 vs 37 ± 11 mL/m2; p = 0.045) and lower ℇCT (7.1 ± 4.6 vs 9.1 ± 3.7; p = 0.05). Post-ablation, patients with AF recurrence had higher LA minimum volume (68 ± 32 vs 55 ± 23; p = 0.05), right atrial volume index (62 ± 20 vs 52 ± 19 mL/m2; p = 0.04) and lower LA active ejection fraction (24 ± 8 vs 29 ± 11; p = 0.05), LA total ejection fraction (39 ± 14 vs 46 ± 12; p = 0.02), LA expansion index (73.6 ± 37.5 vs 94.7 ± 37.1; p = 0.03) and ℇCT (6.2 ± 2.9 vs 7.3 ± 1.7; p = 0.04). Adjusting for clinical variables in the multivariate logistic regression model, post-ablation minimum LA volume (OR 1.09; CI 1.02–1.16), LA expansion index (OR 0.98; CI 0.96–0.99), and baseline ℇR (OR 0.92; CI 0.85–0.99) were independently associated with AF recurrence.

**Conclusion:**

Significant changes in LA volumes and strain parameters occur after AF ablation. CMR derived baseline ℇR, post-ablation minimum LAV, and expansion index are independently associated with AF recurrence.

**Supplementary Information:**

The online version contains supplementary material available at 10.1186/s12968-021-00831-3.

## Background

Atrial fibrillation (AF) is the most common arrhythmia and can have adverse consequences related to reduction in cardiac output and atrial appendage thrombus formation. Affected patients may also be at increased risk for mortality [1, 2]. AF is also a progressive condition that is associated with structural and electrical changes in the left atrium (LA). LA remodelling in AF patients is characterized by LA wall inflammation and fibrosis, an increase in the LA size/volumes, and decrease in LA function [3]. Dynamic changes in the LA volumes during ventricular systole, diastole and atrial contraction are represented as reservoir, conduit and booster/contractile function respectively**.** These LA phasic volumes are influenced by cardiac rhythm and most accurate measurements of LA volumes are performed when patients are in sinus rhythm [4]. These LA parameters can be measured by two dimensional echocardiography (2DE), three dimensional echocardiography (3DE), tissue Doppler imaging, cardiac computed tomography and cardiovascular magnetic resonance (CMR) of which CMR is considered the gold standard [5–8].

For patients with AF in whom a rhythm control strategy is chosen, catheter ablation through pulmonary vein isolation (PVI) is a principal therapeutic intervention to reduce the frequency or eliminate episodes of AF. Some studies have reported that PVI leads to a positive change in LA anatomical and functional parameters which may correlate with AF recurrence [9–11]. CMR strain by tissue tracking is a novel, relatively simple and reproducible technique that is being investigated for assessment of atrial mechanics [5, 12–15]. Standardized strain parameters include LA reservoir strain (ℇR), contractile strain (ℇCT), and conduit strain (ℇCD) [16]. We sought to investigate the association of CMR-derived LA anatomical and functional parameters including strain with AF recurrence post catheter ablation in patients with paroxysmal AF (pAF). We hypothesized that changes in LA function and/or strain as assessed by CMR are associated with AF recurrence after catheter AF ablation.

## Methods

### Patient population

This study was approved by Loyola University Medical Centre’s Institutional Review Board. The inclusion criteria were: (1) patients diagnosed with pAF, (2) underwent a catheter-based PVI between January 2014 and December 2019, (3) underwent pre- and post-ablation CMR. Patients were excluded if they had: (1) implanted pacemaker or implanted cardiovert-defibrillator device, (2) non-sinus rhythm during CMR, (3) were lost to follow up within a year of the ablation procedure, or (4) history of heart transplant. A total of 102 patients met our inclusion criteria. After excluding patients where CMR strain was uninterpretable (n = 15) or those who were lost to follow up (n = 7), 80 patients were included in this analysis. In addition to PVI, patients underwent ablation of LA rotors (n = 13), cavotricuspid isthmus (n = 22), superior vena cava (n = 5) and roof line ablation (n = 8).

### CMR imaging and analysis

CMR was performed prior to (median 32 days; Q1–Q3: 15–67 days) and after (median 131 days; Q1–Q3: 114–201 days) AF ablation. CMR images were acquired using clinically available retrospective electrocardiogram (ECG) gated balanced steady-state free precession sequence on 3 T CMR scanner (Magnetom Trio, Siemens Healthineers, Erlangen, Germany). CMR acquisition parameters included a temporal resolution of ≈50 ms, TR 45 ms, TE 1.2 ms, FOV 300–360 cm, matrix 224 × 204, and slice thickness 7 mm. CMR quantitative analysis was performed on cvi42 (version 5.0; Circle Cardiovascular Imaging Inc., Calgary, Alberta, Canada) using computer assisted manual planimetry. LA volumes were measured using the area–length method (4) and included maximum, minimum, and pre-atrial-contraction volumes from the 2 and 4 chamber views, volume = (0.848 × area4ch × area2ch)/([length2ch + length4ch]/2). All measured LA volumes were indexed to body surface area. The variables of LA volumes (LAV) included in the study were:

- Maximum LA Volume (LAVmax) = LA volume at end systole before mitral valve opening
- Minimum LA Volume (LAVmin) = LA volume at end diastole right after mitral valve closure
- Preatrial Contraction LA volume (LAVPreA) = LA volume before atrial contraction

Using the measured LA volumes at different points of cardiac cycle, LA ejection fraction (LAEF) was calculated as follows:

- Passive LAEF: (LAVmax−LAVPreA)/LAVmax.
- Active LAEF: (LAVPreA−LAVmin)/LAVPreA.
- Total LAEF: (LAVmax−LAVmin)/LAVmax.
- Expansion index: (LAVmax−LAVmin)/LAVmin

**Fig. 1:**
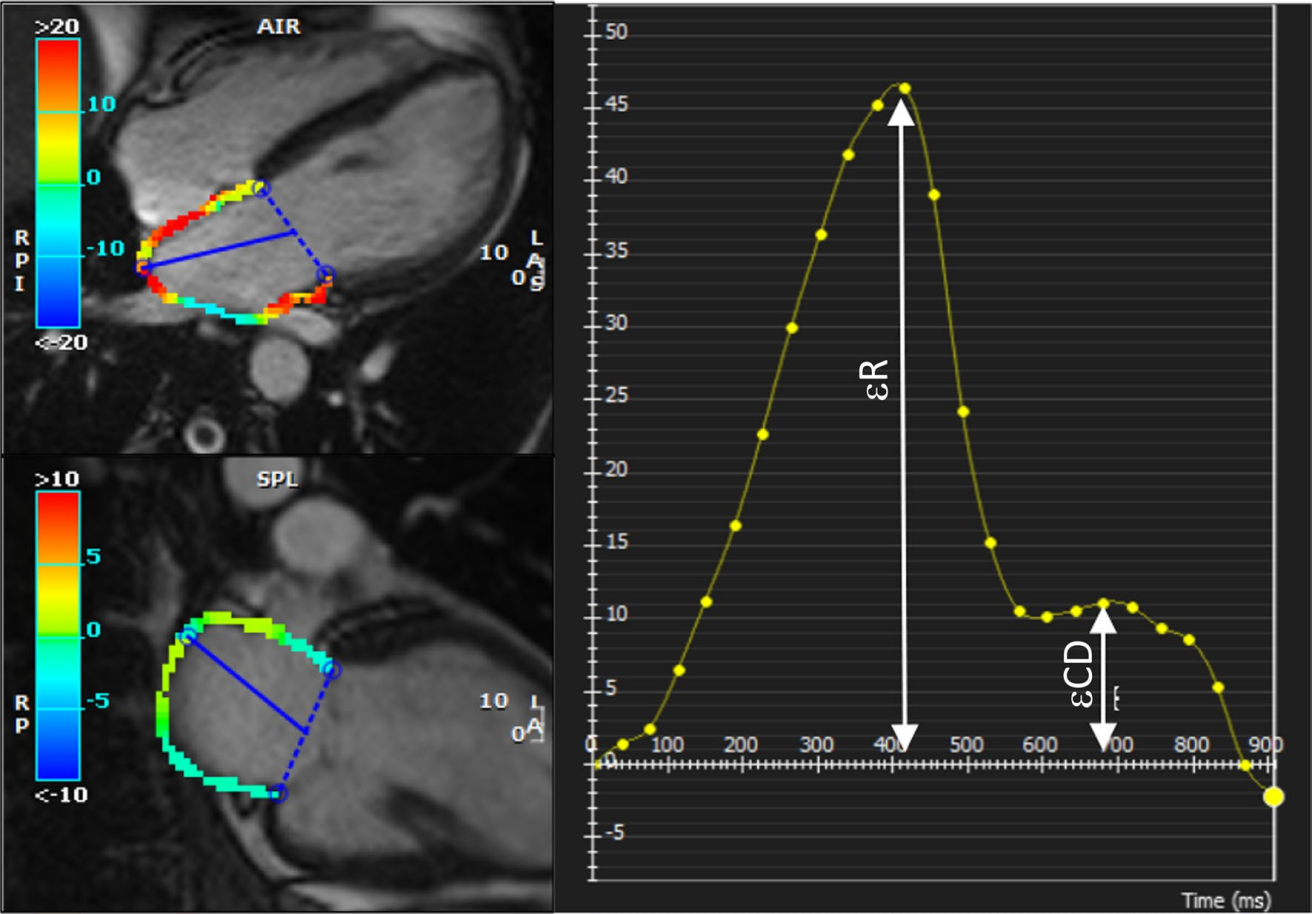
Left: Example of left atrial (LA) tissue tracking in still frames of cine cardiovascular magnetic resonance (CMR) (balanced steady-state free precession imaging) four chamber (left top) and two chamber (left bottom) cine loops. Right: Longitudinal strain with two peaks representing reservoir (ℇR) and contractile (ℇCT) strain. The difference between the two measurements is conduit strain (ℇCD)

Multimodality Tissue Tracking software (version 5.0, Circle Cardiovascular Imaging Inc.) was used to measure ℇR and ℇCT from 4- and 2-chamber cine CMR images (Fig. 1), the difference between these two measurements was ℇCD [16]. Strain measurements were performed by computer assisted manual planimetry where the investigator defines endocardial and epicardial borders of the LA at end systole and using the marked points, the software creates endocardial and epicardial borders and then tracks LA wall in subsequent frames. CINE loops with superimposed longitudinal strain tracings were played to confirm accurate tracking of the LA endo- and epicardium throughout the cardiac cycle. If tracking was inaccurate at some point during the cardiac cycle, the planimetry was corrected manually and the software algorithm reapplied. The final strain results were taken as an average of the 2 and 4 chamber views. This method has been previously validated with excellent reproducibility (intraclass correlation coefficients between 0.90 and 0.97 for LA volumes and strain) [16–18]. Strain measurements were accepted only in the case of adequate tracking quality in at least five of the six segments per view. We also calculated ΔℇR, ΔℇCT, ΔℇCD as the difference between pre- and post-ablation values of ℇR, ℇCT and ℇCD, respectively. Strain measurements were performed by a CMR trained and board-certified investigator with one year experience who was blinded to the case status of the participants. All patients were followed up in outpatient clinic for monitoring and arrhythmia management. AF recurrence was identified by ECG documentation of an atrial tachyarrhythmia lasting ≥ 30 s on a 12-lead ECG, event recording, or Holter monitor recording. Recurrence rates were determined regardless of whether patients were on antiarrhythmic medications or not. Atrial arrhythmias that occurred during the first 90 days after catheter ablation and resolved spontaneously or with cardioversion, were not counted as recurrences (blanking period).

### Statistical analysis

Continuous variables are presented as mean ± SD if normally distributed and as medians with interquartile ranges (Q1–Q3) if not normally distributed. Categorical variables are presented as frequencies and percentages. Differences between group means were evaluated with t tests for continuous variables or χ2 analysis for categorical variables. Multivariate logistic regression model was constructed adjusting for the following clinical parameters: age, gender, hypertension, obstructive sleep apnea in addition to the CMR-derived LA parameters with P values < 0.1. To avoid collinearity, correlations between continuous variables were tested using the Spearman correlation coefficient and variables with r > 0.50 were not included in the model. We used a modified Cox proportional hazard model to estimate the time-dependent hazard ratios of AF recurrence in tertiles of patients based on baseline ℇR and post-ablation ℇCT. Cox models were adjusted for the same predictors of AF recurrence as used for the multivariable model, mentioned above. Statistical analyses were performed using MedCalc for Windows (version 15.0, MedCalc Software, Ostend, Belgium). All P values reported are from two-sided tests, and P values < 0.05 were considered statistically significant.

## Results

Baseline demographics, clinical characteristics and medications at the time of the ablation procedure are listed in Table 1. Mean age was 58.6 ± 9.4 years, 75% were men, mean CHA_2_DS_2_-VASc score was 1.7 ± 1.4; 36% had prior cardioversion and 51% were taking antiarrhythmic drugs. Patients were followed for a median of 4 years (Q1–Q3 = 2.5–6.2 years) after AF ablation. Of 80 patients, 21 (26.3%) patients had AF recurrence. The median duration after catheter ablation to AF recurrence was 5 months (Q1–3: 3–11 months). There were no significant differences between AF recurrence vs. no AF recurrence groups in age, gender, CHA_2_DS_2_-VASc score, or baseline comorbidities (all P > 0.05).

**Table 1:** Patient baseline characteristics

	Overall n = 80	No AF recurrence n = 59	AF recurrence n = 21	p-value
Demographics				
Age, mean ± SD	58.6 ± 9.4	58.5 ± 11.5	58.6 ± 8.9	0.99
Male	60 (75)	42 (71.2)	18 (85.7)	0.2
Caucasian	76 (95)	54 (91.5)	21 (100)	0.64
Prior DC cardioversion	29 (36.3)	23 (39)	6 (28.6)	0.2
Comorbidities				
Current/former smoker	20 (25)	16 (27.1)	4 (19)	0.29
Coronary artery disease	10 (12.5)	9 (15.3)	1 (4.8)	0.16
Peripheral arterial disease	1 (1.25)	0	1 (4.8)	0.12
Heart failure	8 (10)	7 (11.9)	1 (4.8)	0.28
Hypertension	38 (47.5)	27 (45.8)	11 (52.4)	0.92
Obstructive sleep apnea	18 (22.5)	11 (18.6)	7 (33.3)	0.33
Diabetes mellitus	3 (3.8)	3 (5.1)	0	0.27
Cerebrovascular disease	1 (1.25)	1 (1.7)	0	0.53
CHA_2_DS_2_-VASc score				
0	22 (27.5)	16 (27.1)	6 (28.6)	0.79
1	32 (40)	21 (35.6)	11 (52.4)	0.45
2	16 (20)	13 (22)	3 (14.3)	0.30
≥ 3	10 (12.5)	7 (11.9)	3 (14.3)	0.61
Medications				
Aspirin	17 (21.3)	11 (18.6)	6 (28.6)	0.57
Statin	29 (36.3)	1 (1.7)	2 (9.5)	0.15
Warfarin	9 (11.3)	7 (11.9)	2 (9.5)	0.62
Apixaban	12(15)	9 (15.3)	3 (14.3)	0.73
Rivaroxaban	35(43.8)	23 (39.0)	12 (57.1)	0.43
Dabigatran	4 (5)	3 (5.1)	1 (4.8)	0.85
Beta Blocker	55 (68.8)	40 (67.8)	15 (71.4)	0.52
Calcium channel blocker	22 (27.5)	18 (30.5)	4 (19)	0.18
Anti-Arrhythmic Therapy	45 (56.3)	32 (54.2)	13 (61.9)	0.33
Flecanaide	17 (21.3)	10 (16.9)	7 (33.3)	0.25
Propafenone	4 (5)	3 (5.1)	1 (4.8)	0.85
Sotalol	4 (5)	1 (1.7)	3 (14.3)	**0.04**
Amiodarone	4 (5)	4 (6.8)	0	0.20
Dronderone	14 (17.5)	11 (18.6)	3 (14.3)	0.48
ACE/ARB	22 (27.5)	19 (32.2)	3 (14.3)	0.06
Levothyroxine	7 (8.8)	4 (6.8)	3 (14.3)	0.42
Diuretic	14 (17.5)	11 (18.6)	3 (14.3)	0.48

Significant p-values are shown in bold

ACE, angiotensin converting enzyme inhibitor; ARB, angiotensin receptor blocker; DC, direct current

AF ablation was associated with a decrease in LA volumes (LAVmax, LAVmin, LAVpreA), pulmonary vein ostial diameters and all three LA strain parameters (Table 2). No significant improvement was seen in right atrial (RA) volumes or left ventricular (LV) and right ventricular (RV) volumes and ejection fraction.

**Table 2:** Comparison of pre and post ablation LA characteristics on CMR

	Pre-ablation	Post-ablation	p-value
LAV maximum (mL)	112 ± 36	96 ± 30	**0.003**
LAV maximum index (mL/m^2^)	52 ± 18	45 ± 14	**0.01**
LAV minimum (mL)	63 ± 32	53 ± 26	**0.03**
LAV minimum index (mL/m^2^)	29 ± 16	25 ± 12	**0.046**
LAV pre-A (mL)	86 ± 35	73 ± 27	**0.007**
LAV pre-A index (mL/m^2^)	40 ± 17	34 ± 13	**0.02**
LA passive EF (%)	25 ± 12	24 ± 9	0.59
LA active EF (%)	29 ± 15	27 ± 16	0.31
LA total EF (%)	46 ± 15	43 ± 15	0.25
LA expansion index	95 ± 51	88 ± 49	0.41
LVEF	60 ± 9	57 ± 6	0.1
LVEDVI (mL/m^2^)	86 ± 16	90 ± 25	0.33
LVESVI (mL/m^2^)	36 ± 11	39 ± 11	0.1
LV mass (mg)	97 ± 33	88 ± 27	0.07
RVEF	53 ± 6	51 ± 6	0.09
RVEDVI (mL/m^2^)	94 ± 22	93 ± 22	0.89
RVESVI (mL/m^2^)	45 ± 14	46 ± 13	0.48
RA volume (mL)	143 ± 40	132 ± 39	0.07
RA volume index	68 ± 19	65 ± 19	0.32
RUPV diameter (mm)	19.5 ± 3.2	16.9 ± 3.8	** < 0.001**
RLPV diameter (mm)	17.9 ± 3.1	16.6 ± 3.1	**0.01**
LUPV diameter (mm)	15.2 ± 2.5	12.9 ± 3.2	** < 0.001**
LLPV diameter (mm)	13.9 ± 2.1	11.7 ± 4.5	**0.03**
ℇR	24.0 ± 10.4	19.8 ± 8.1	**0.005**
ℇCT	8.4 ± 0.1	5.7 ± 5.3	**0.001**
ℇCD	15.6 ± 7.1	13.0 ± 6.3	**0.02**

Significant p-values are shown in bold

EDV, end diastolic volume; EF, ejection fraction; ESV, end systolic volume; LA, left atrium; LAV, left atrial volume; LLPV, left lower pulmonary vein; LUPV, left upper pulmonary vein; LV, left ventricle; LVEDVI, left ventricular end-diastolic volume index; LVESVI, left ventricular end-systolic volume index; RA, right atrium; RLPV, right lower pulmonary vein; RUPV, right upper pulmonary vein; RV, right ventricle; RVEDVI, right ventricular end-diastolic volume index; RVESVI, right ventricular end-systolic volume index; ℇCD, conduit strain; ℇCT, contractile strain; ℇR, reservoir strain

At baseline, patients with AF recurrence (Table 3) had lower LV end systolic volume index (p = 0.045) and lower ℇCT (p = 0.05). Post-ablation, patients with AF recurrence had higher LA minimum volume (p = 0.05), RA volume index (p = 0.04) and lower LA active ejection fraction (p = 0.05), LA total ejection fraction (p = 0.02), LA expansion index (p = 0.03) and ℇCT (p = 0.04).

**Table 3:** Pre- and post-ablation CMR characteristics of patients with no AF recurrence versus those with AF recurrence

	Total N = 80	No AF recurrence n = 59	AF recurrence n = 21	p-value
Pre-ablation				
CMR before ablation procedure (median days, Q1-Q3)	32 (15–67)	26 (12–64)	38 (99–306)	0.45
LAV maximum (mL)	112 ± 36	110 ± 34	117 ± 43	0.45
LAV maximum index (mL/m^2^)	52 ± 18	46 ± 16	47 ± 16	0.51
LAV minimum (mL)	63 ± 32	60 ± 30	70 ± 38	0.21
LAV minimum index (mL/m^2^)	29 ± 16	28 ± 16	33 ± 16	0.24
LAV pre-A (mL)	86 ± 35	82 ± 33	93 ± 41	0.21
LAV pre-A index (mL/m^2^)	40 ± 17	38 ± 18	43 ± 17	0.24
LA passive ejection fraction (%)	25 ± 12	23 ± 9	24 ± 8	0.71
LA active ejection fraction (%)	29 ± 15	30 ± 16	26 ± 14	0.30
LA total ejection fraction (%)	46 ± 15	45 ± 11	43 ± 13	0.47
LA expansion index	95 ± 51	168 ± 524	87 ± 58	0.46
LV ejection fraction (%)	60 ± 9	60 ± 10	61 ± 4	0.93
LVEDVi (mL/m^2^)	86 ± 16	82 ± 17	88 ± 15	0.11
LVESVi (mL/m^2^)	36 ± 11	32 ± 7	37 ± 11	**0.05**
LV mass (mg)	97.0 ± 33.4	95.1 ± 32.3	102.4 ± 36.7	0.38
RVEF (%)	53 ± 6	52 ± 6	55 ± 7	0.12
RVEDVi (mL/m^2^)	94 ± 22	95 ± 22	91 ± 24	0.55
RVESVi (mL/m^2^)	45 ± 14	44 ± 10	45 ± 22	0.89
RA volume (mL)	143 ± 40	139 ± 38	155 ± 48	0.12
RAVI (mL/m^2^)	68 ± 19	67 ± 19	70 ± 20	0.55
ℇR	24.0 ± 10.4	25.2 ± 10.7	20.8 ± 8.9	0.06
ℇCT	8.4 ± 4.1	9.1 ± 3.7	7.1 ± 4.6	**0.05**
ℇCD	15.6 ± 7.1	16.2 ± 7.7	13.7 ± 4.9	0.17
Post-ablation				
CMR after ablation procedure (median days, Q1–Q3)	131 (114–201)	140 (99–306)	124 (96–151.5)	0.31
LA maximum volume (mL)	96 ± 30	100 ± 27	104 ± 36	0.57
LAV maximum index (mL/m^2^)	45 ± 14	47 ± 14	47 ± 16	0.85
LAV minimum (mL)	53 ± 23	55 ± 22	68 ± 26	**0.05**
LAV minimum index (mL/m^2^)	25 ± 12	26 ± 8	31 ± 11	0.06
LAV pre-A (mL)	73 ± 27	77 ± 25	79 ± 32	0.74
LAV pre-A index (mL/m^2^)	34 ± 13	37 ± 12	36 ± 14	0.79
LA passive EF (%)	24 ± 9	24 ± 9	24 ± 8	0.96
LA active EF (%)	27 ± 16	29 ± 11	24 ± 8	**0.05**
LA total EF (%)	43 ± 15	46 ± 12	39 ± 14	**0.02**
LA expansion index	88 ± 49	95 ± 37	74 ± 38	**0.03**
LVEF (%)	57 ± 6	58 ± 6	60 ± 3	0.85
LVEDVI (mL/m^2^)	90 ± 25	85 ± 19	88 ± 38	0.79
LVESVI (mL/m^2^)	39 ± 11	36 ± 12	34 ± 8	0.77
LV mass (mg)	88.2 ± 27.0	94.2 ± 26.6	96.1 ± 28.8	0.69
RVEF (%)	51 ± 6	52 ± 6	53 ± 5	0.42
RVEDVI (mL/m^2^)	93 ± 22	89 ± 21	87 ± 24	0.86
RVESVI (mL/m^2^)	46 ± 13	43 ± 13	39 ± 11	0.81
RA volume (mL)	132 ± 39	121 ± 38	137 ± 42	0.11
RAVI (mL/m^2^)	65 ± 19	52 ± 18	62 ± 20	**0.04**
ℇR	21.4 ± 8.1	21.4 ± 7.8	21.0 ± 9.1	0.77
ℇCT	7.0 ± 5.3	7.3 ± 1.7	6.2 ± 2.9	**0.04**
ℇCD	14.4 ± 6.3	14.1 ± 6.2	14.8 ± 7.2	0.3
ΔℇR	2.7 ± 9.3	3.8 ± 9.1	-0.2 ± 9.7	0.09
ΔℇCT	1.4 ± 4.5	1.7 ± 4.2	0.9 ± 5.3	0.49
ΔℇCD	1.3 ± 6.6	2.1 ± 6.7	-1.1 ± 6.2	0.06

Significant p-values are shown in bold

EDV, end diastolic volume; EF, ejection fraction; ESV, end systolic volume; LA, left atrium; LAV, left atrial volume; LLPV, left lower pulmonary vein; LUPV, left upper pulmonary vein; LV, left ventricle; RA, right atrium; RLPV, right lower pulmonary vein; RUPV, right upper pulmonary vein; RV, right ventricle; ℇCD, conduit strain; ℇCT, contractile strain; ℇR, reservoir strain, ΔℇR, ΔℇCT, ΔℇCD, difference between pre- and post-ablation values of ℇR, ℇCT and ℇCD, respectively

**Fig. 2:**
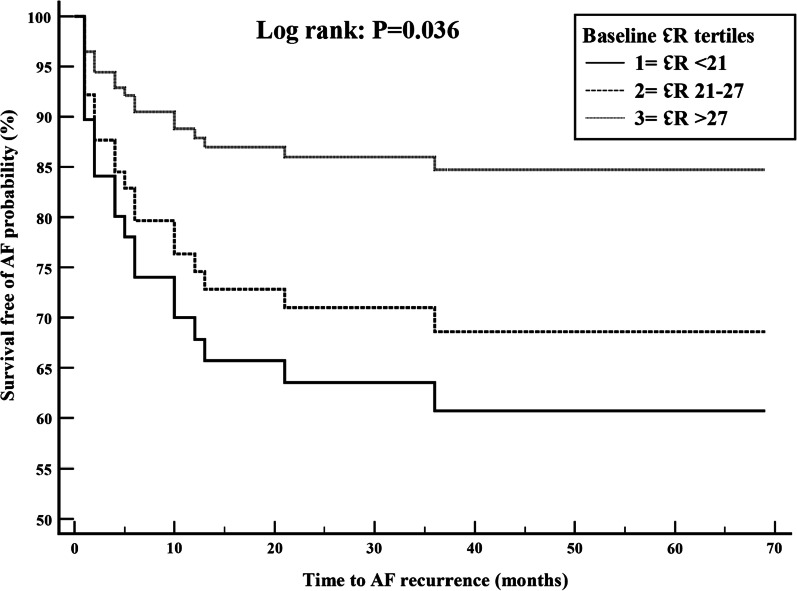
Cox-proportional hazard model for survival free of atrial fibrillation by tertiles of baseline reservoir strain (ℇR), adjusted for age, gender, hypertension, and obstructive sleep apnea. Tertiles one, two, three corresponded to a baseline ℇR of < 21, 21–27 and > 27, respectively

**Fig. 3:**
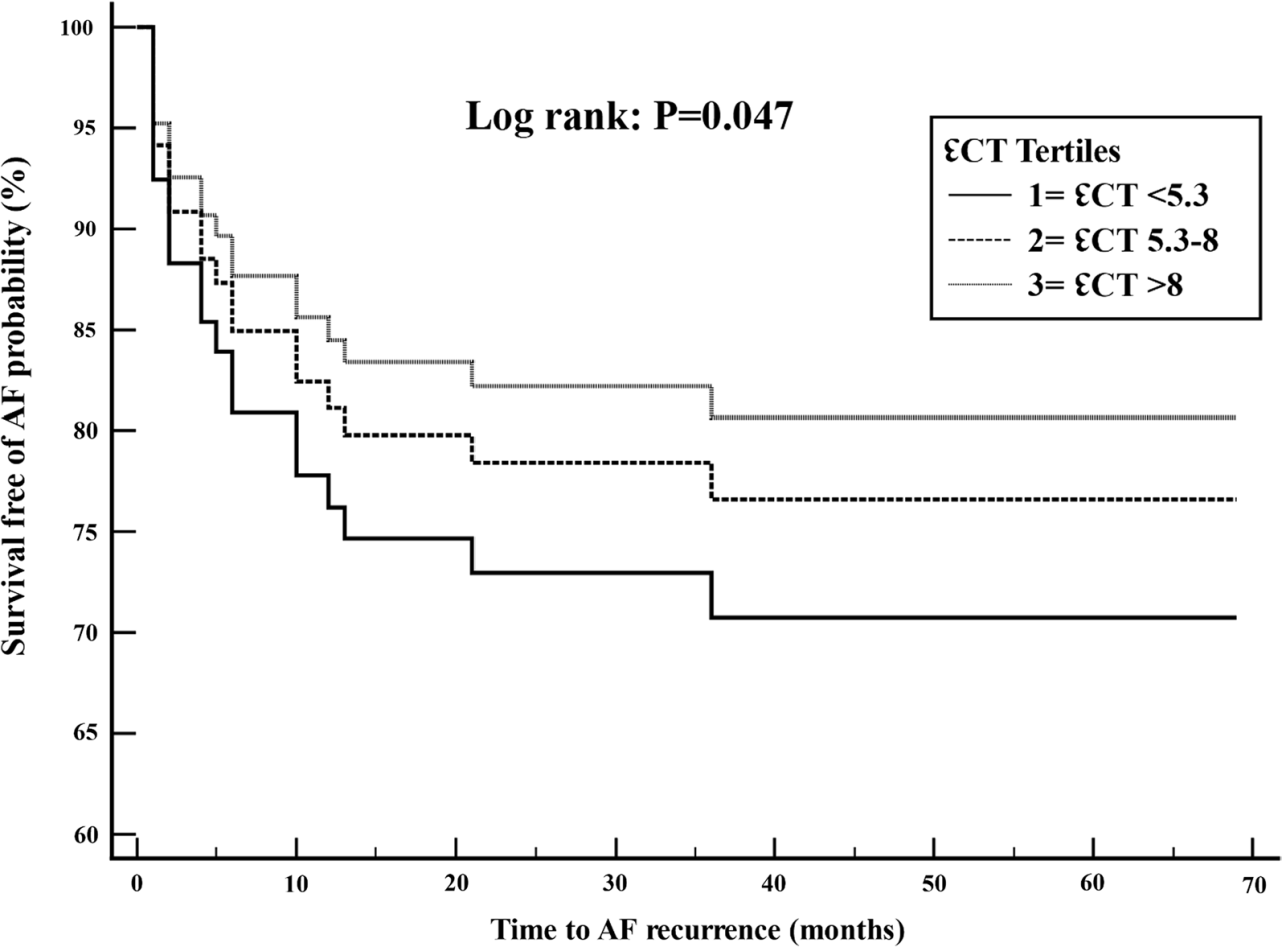
Cox-proportional hazard model for survival free of atrial fibrillation by tertiles of post-ablation contractile strain (ℇCT), adjusted for age, gender, hypertension, and obstructive sleep apnea. Tertiles one, two, three corresponded to a post-ablation ℇCT of < 5.3, 5.3–8 and > 8, respectively

Adjusting for clinical variables in the multivariate logistic regression model, post-ablation minimum LAV (OR 1.09; CI 1.02–1.16), LA expansion index (OR 0.98; CI 0.96–0.99), and baseline ℇR (OR 0.92; CI 0.85–0.99) were independently associated with AF recurrence. (Table 4) Cox proportional hazard model results are presented in Figs. 2 and 3 where survival free of AF is represented for the three tertiles of the baseline ℇR and post-ablation ℇCT. For the baseline ℇR model, tertiles one, two, and three corresponded to a baseline ℇR of < 21.5, 21.5–27 and > 27, respectively. The model showed a longer survival free of AF in patients with a higher baseline ℇR. For the post-ablation ℇCT model, teriles one, two, and three corresponded to a post-ablation ℇCT of < 5.3, 5–8 and > 8, respectively. The model showed a trend towards longer survival free of AF in patients with higher post-ablation ℇCT. Additional file 1 shows comparison graphs of baseline and post-ablation reservoir and contractile strain in AF recurrence and no-recurrence groups.

**Table 4:** Univariate (unadjusted) and multi-variate (adjusted) logistic regression results for factors associated with AF recurrence

Variable	Unadjusted odds ratio	Confidence interval	P value	Adjusted odds ratio	Confidence interval	P value
Age	1.01	0.96–1.06	0.57	1.03	0.95–1.09	0.47
Female gender	2.4	0.6–9.3	0.29	1.9	0.49–6.7	0.33
Hypertension	1.15	0.43–3.13	0.44	1.19	0.57–2.58	0.26
Obstructive sleep apnea	1.96	0.61–6.29	0.09	3.2	0.68–15.01	0.07
LAVmax (post-ablation)	1.00	0.95–1.02	0.19	0.94	0.89–1.0	0.05
LAVmin (post-ablation)	1.02	1.00–1.04	0.06	1.09	1.02–1.16	**0.01**
LA expansion index (post-ablation)	0.98	0.97–0.99	**0.04**	0.98	0.96–0.99	**0.02**
Baseline ℇR	0.94	0.86–0.99	**0.04**	0.92	0.85–0.99	**0.03**
Post-ablation ℇR	1.02	0.95–1.07	0.41			
Baseline ℇCT	0.91	0.83–0.99	**0.03**	0.95	0.67–1.13	0.06
Post-ablation ℇCT	0.88	0.76–0.99	0.05	0.84	0.66–1.07	0.08

Significant p-values are shown in bold

LA, left atrium; LAV, left atrial volume; RA, right atrium; ℇR, reservoir strain; ℇCT, contractile strain

## Discussion

In this retrospective study, we evaluated the association of CMR derived LA anatomical and functional parameters in pAF patients with AF recurrence after catheter ablation. Our main finding were that: (1) PVI leads to a decrease in LA volumes with a decrease in all LA strain parameters (2) baseline ℇR and post-ablation minimum LAV and LA expansion index are independently associated with AF recurrence.

AF recurrence is not uncommon after catheter ablation with freedom from atrial arrhythmia at long-term follow-up (≥ 2 years) after a single procedure is about 53% which increases to 80% with multiple procedures [19]. Early recurrence is thought to be due to leaking of AF electrical impulses due to less early fibrous tissue formation around pulmonary veins after PVI whereas late recurrence is more likely from the shifting of AF foci from pulmonary veins to the LA due to stressors and other co-morbid conditions [20]. Poor LA function has been associated with several poor outcomes including implantable cardioverter defibrillator (ICD) shocks [17, 18, 21–23], ischemic events and worsening LV diastolic function in patients [22]. LA size and volumes have been reported as predictors of sinus rhythm maintenance after AF ablation [24, 25]. LA size using echocardiographic derived anteroposterior LA diameter has been studied mostly. A meta-analysis by Zhuang et al. of 22 studies (3750 subjects) that used anteroposterior LA diameter showed that the mean difference of LA diameter between patients with and without recurrence was 1.9 mm (95% CI 1.3–2.5, P < 0.001) and dilated LA was associated with significantly increased risk of AF recurrence after single PVI [24]. LAV is more accurate in assessing LA size but studies using LAV as a predictor of AF recurrence have been contradictory. Njoku et al. published a meta-analysis of 21 studies (3822 subjects) that used LAV/LAV index as a predictor of AF recurrence after PVI [25]. Both mean LAV (11 studies, 1559 subjects) and LAV index (9 studies, 1425 subjects) were higher in patients with recurrence (OR 1.03, CI 1.01–1.05). Our study had a more comprehensive design and evaluated both volumes and LA function parameters pre and post ablation as predictors of AF recurrence after PVI.

LAV measured in our study were higher than normal values by CMR reported in the literature [26]. Considering that our study only included patients with pAF, this is not an unexpected finding. Our LAV were fairly similar to previous studies in patients with AF [9, 10]. LA remodelling may occur after PVI and entails a decrease in LAV and an increase in LAEF [10]. This LA remodelling has been associated with AF recurrence, more so with late than early recurrence [27]. Recent data from Multi-Ethnic Study of Atherosclerosis (MESA) study showed that adverse remodelling i.e. elevated LAV and decreased passive and total LAEF, precedes AF in a multi-ethnic population who were free of clinical cardiovascular disease at baseline [26].

LA strain is a highly reproducible [16] measure of LA deformation and has been shown to be a sensitive marker of intracavitary pressures and LA reservoir function [28]. LA strain is also a useful marker of AF risk incremental to LA size in the general population and is an independent predictor of AF recurrence [28, 29].

In our study, baseline ℇR was significantly associated with AF recurrence in the multivariate analysis. This is in line with previous echocardiographic studies that showed an association between the baseline strain and AF recurrence following PVI or cardioversion [12, 30]. We also found a significant association of post ablation LAVmin and expansion index with AF recurrence after PVI, however, the association was stronger with baseline ℇR than volumetric indices. Baseline ℇR would also be more relevant clinically to help risk stratify patients prior to ablation. Studies using echocardiography have shown that functional parameters of LA strain and strain rate on tissue Doppler imaging or speckle echocardiography are better predictors of AF recurrence than LAV [30–33]. Theoretically, LA strain is less affected by loading conditions and by tethering effects than volumetric measurements [22]. Our study extends these findings to CMR as a potential tool for estimating future risk of AF recurrence by using ℇR prior to ablation.

## Limitations

Several limitations need to be considered when interpreting the results of our study. Our study is a ret r osp ective observational study, with some inherent biases related to the study design including a lack of standardization in patient follow up clinic visits and rhythm monitoring strategies to detect AF recurrence. As described in the methods section, some patients underwent additional ablation in addition to the standard PVI and that might have affe cted the post-ablation LA anatomic and functional parameters. We only included pAF patients who have undergone radiofrequency ablation. Our results m i ght n ot be generalizable to those with persistent AF or those who underwent other forms of ablation, e.g. cryoablation. Due to the relatively small sample size, some of the variables may not have reached statistical significance. Although post-ablation CMR is a common routine practice at our institution, selection bias might be significant as we only included patients who were in sinus rhythm at the time of pre and post-ablation CMR. We used clinically available CMR cine sequence with a temporal resolution of 50 ms for strain measurements based on previously published data by MESA investigators [21]. A higher temporal resolution might have produced more precise measurements. There is no dedicated validated CMR software exclusively for LA strain measurements. We utilized the strain software developed for LV strain as done by other investigators. LA late gadolinium enhancement noted on CMR is an important parameter of LA fibrosis that might have a prognostic factor for AF recurrence, however, it was not investigated in this study.

## Conclusions

We investigated structural and functional parameters of LA in patients with pAF undergoing PVI and found that baseline ℇR and post-ablation minimum LAV and expansion index were independently associated with AF recurrence. Among these parameters, baseline ℇR would be more clinically useful as most patients don’t routinely undergo post ablation CMR. Patients with a higher risk of recurrence might need closer surveillance, more intense risk factor modification and/or modification of their antiarrhythmic regimen. Further validation studies are needed before CMR parameters can be included in the routine risk models in AF patients.

## Supplementary Information

**Additional file 1:** Multiple comparison graphs of the baseline and post-ablation reservoir (top) and contractile (bottom) strain data in both groups.

## Data Availability

The datasets used and/or analysed during the current study are available from the corresponding author on reasonable request.

## Abbreviations

ℇCD: Conduit strain
ℇCT: Contractile strain
ℇR: Reservoir strain
2DE: Two dimensional echocardiography
3DE: Three dimensional echocardiography
AF: Atrial fibrillation
CMR: Cardiovascular magnetic resonance
ECG: Electrocardiography
LA: Left atrium
LAEF: Left atrial ejection fraction
LAV: Left atrial volume
LAVmax: Maximal left atrial volume
LAVmin: Minimum left atrial volume
LAVpreA: Pre atrial systole left atrial volume
LV: Left ventricle/left ventricular
LVEDVI: Left ventricular end-diastolic volume index
LVESVI: Left ventricular end-systolic volume index
pAF: Paroxysmal atrial fibrillation
PVI: Pulmonary vein isolation
RA: Right atrium/right atrial
RV: Right ventricular/right ventricular
RVEDVI: Right ventricular end-diastolic volume index
RVESVI: Right ventricular end-systolic volume index
