# Supplementary material for: Association of left atrial strain by cardiovascular magnetic resonance with recurrence of atrial fibrillation following catheter ablation
Source: J Cardiovasc Magn Reson. 2022 Jan 3;24:3. doi: 10.1186/s12968-021-00831-3 (PMC8722067; doi:10.1186/s12968-021-00831-3)

**Supplemental material:** Multiple comparison graphs of the baseline and post-ablation reservoir (top) and contractile (bottom) strain data in both groups


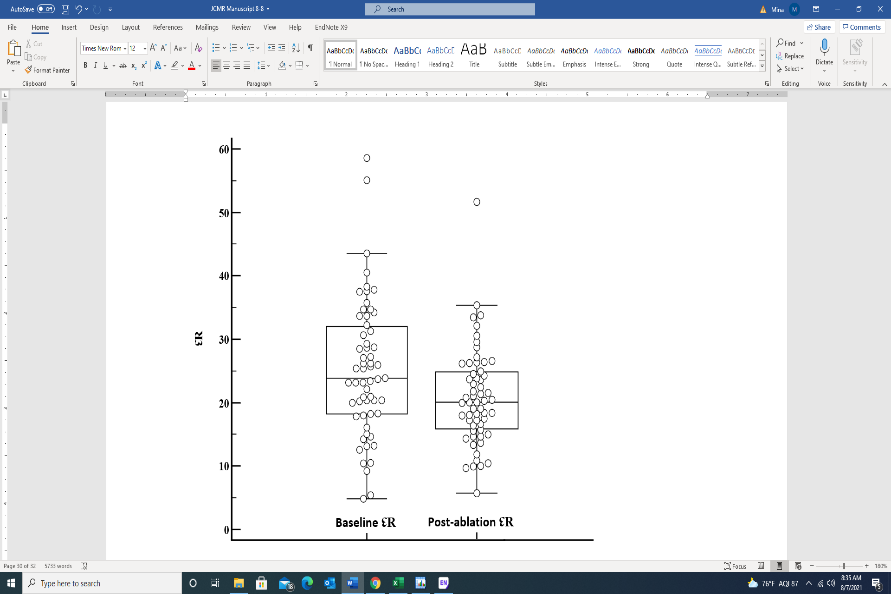

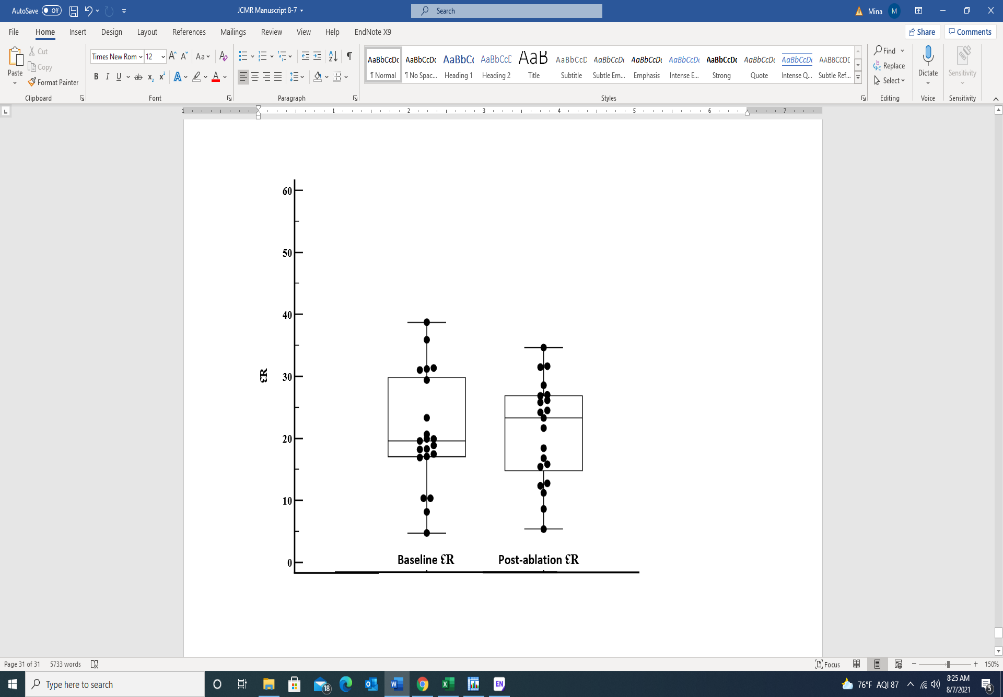

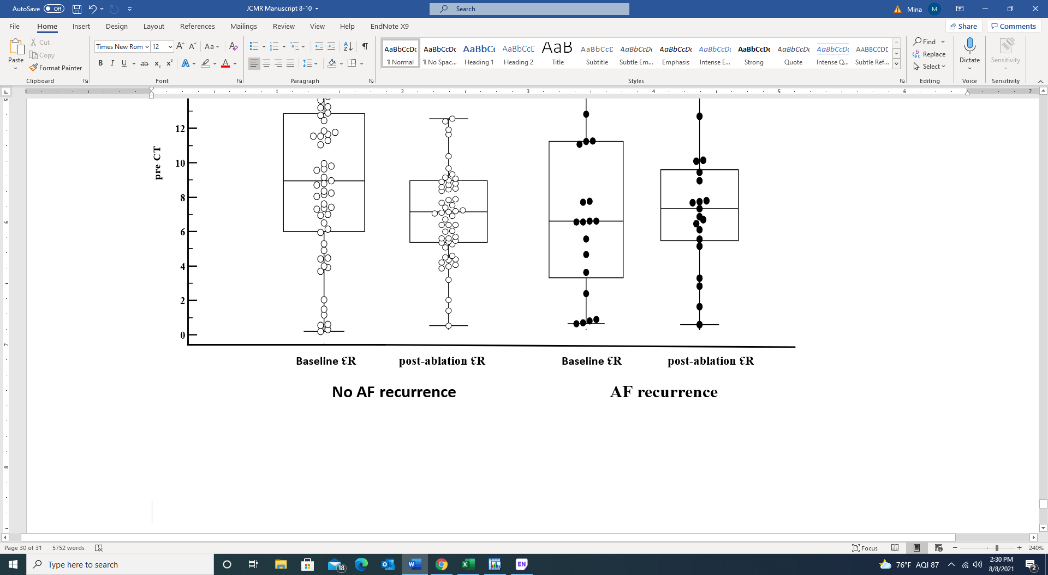

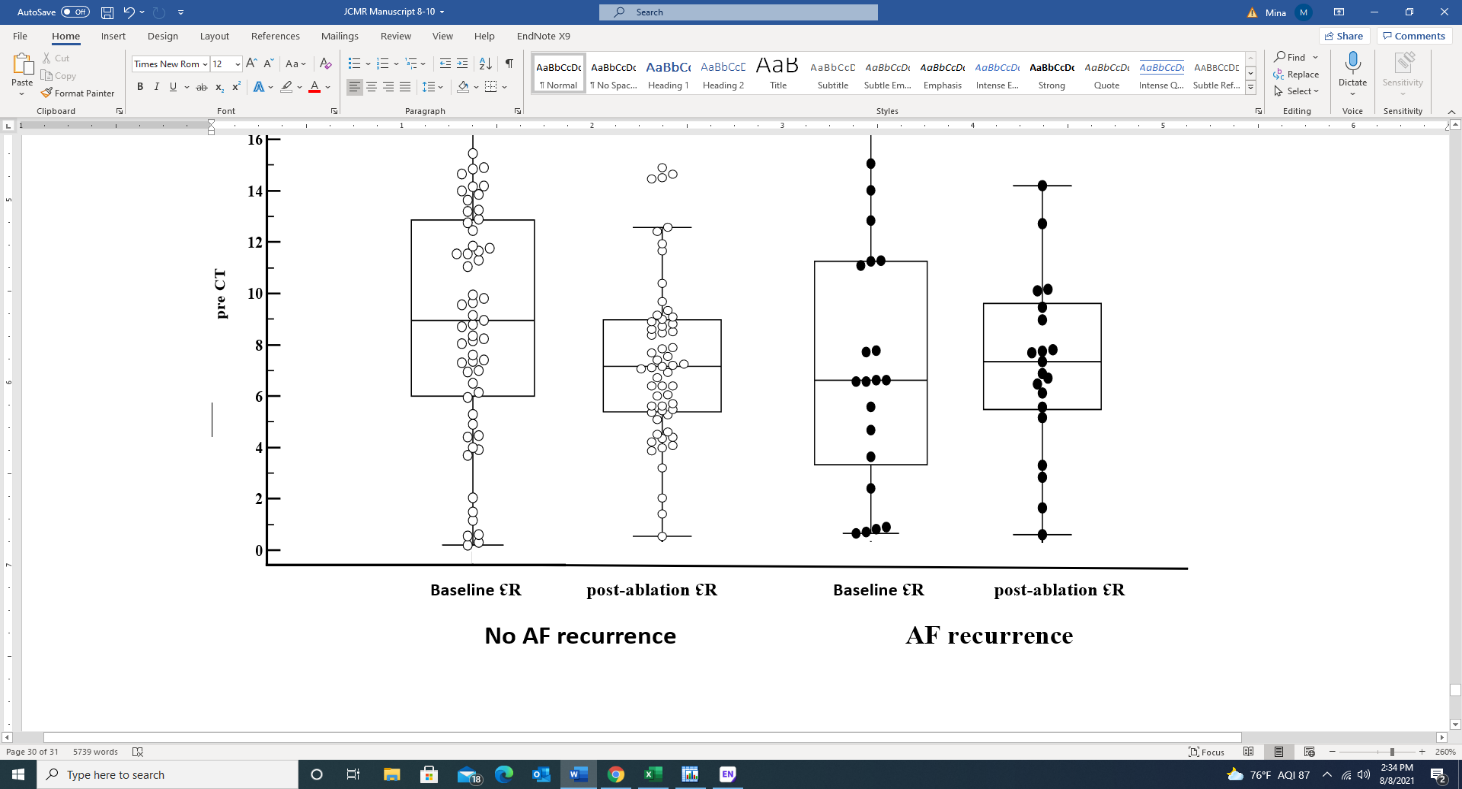


**Baseline ↋CT post-ablation ↋CT Baseline ↋CT post-ablation ↋CT**


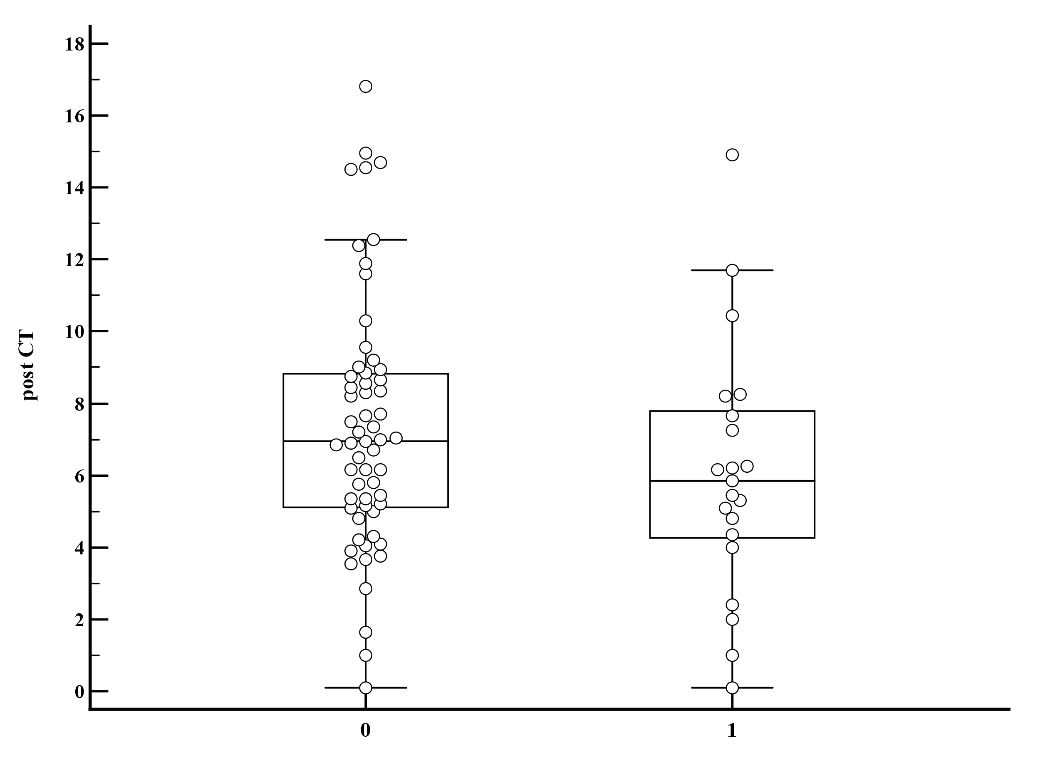

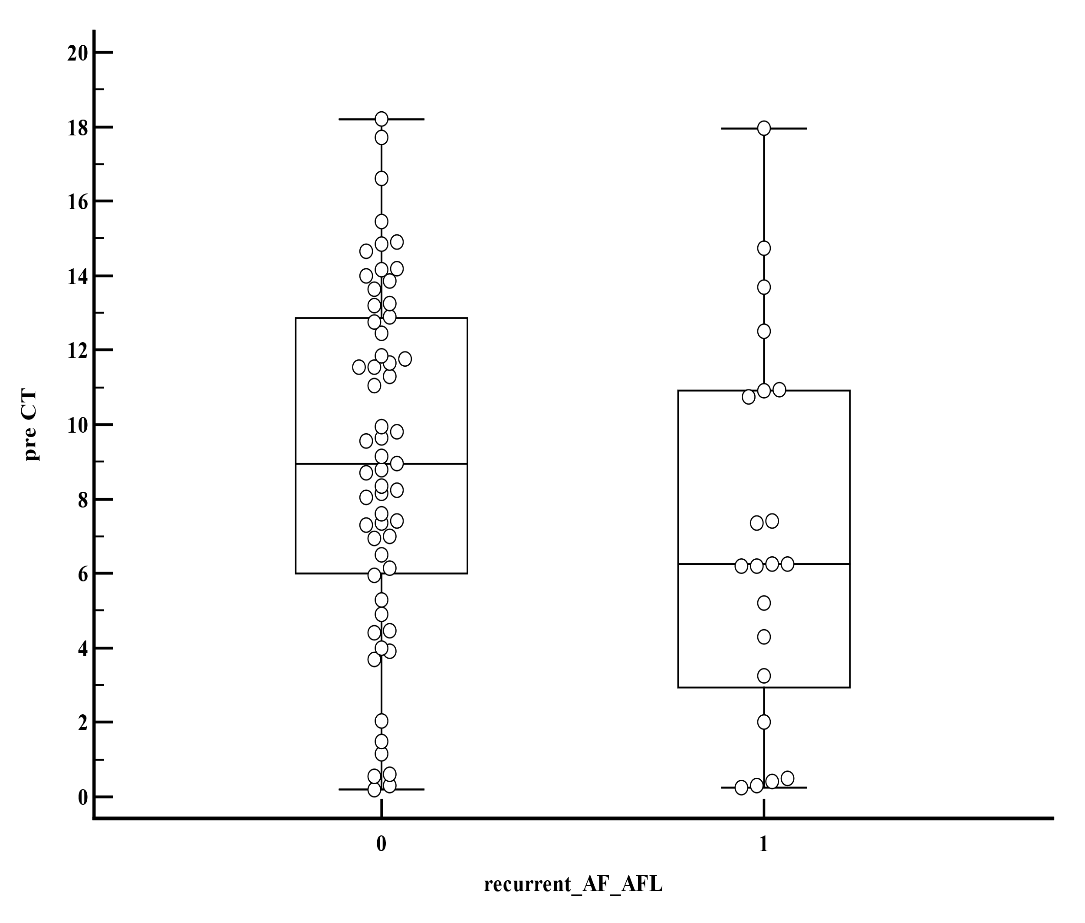


**No AF recurrence AF recurrence**


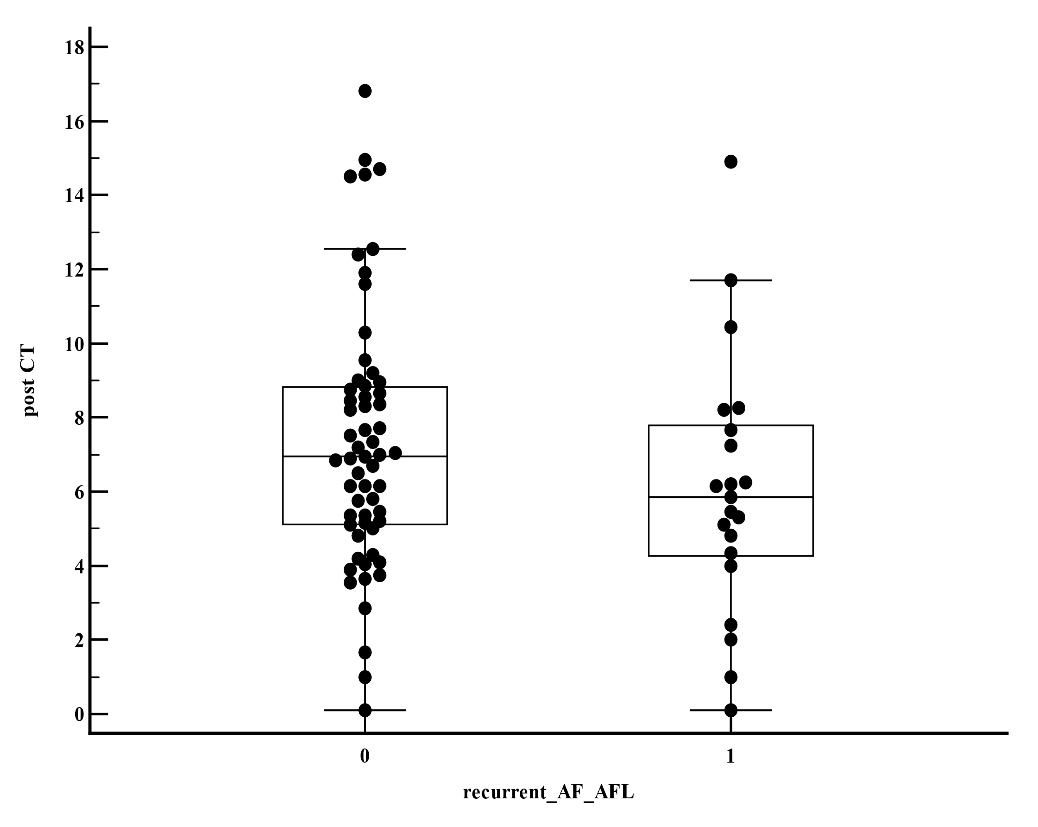

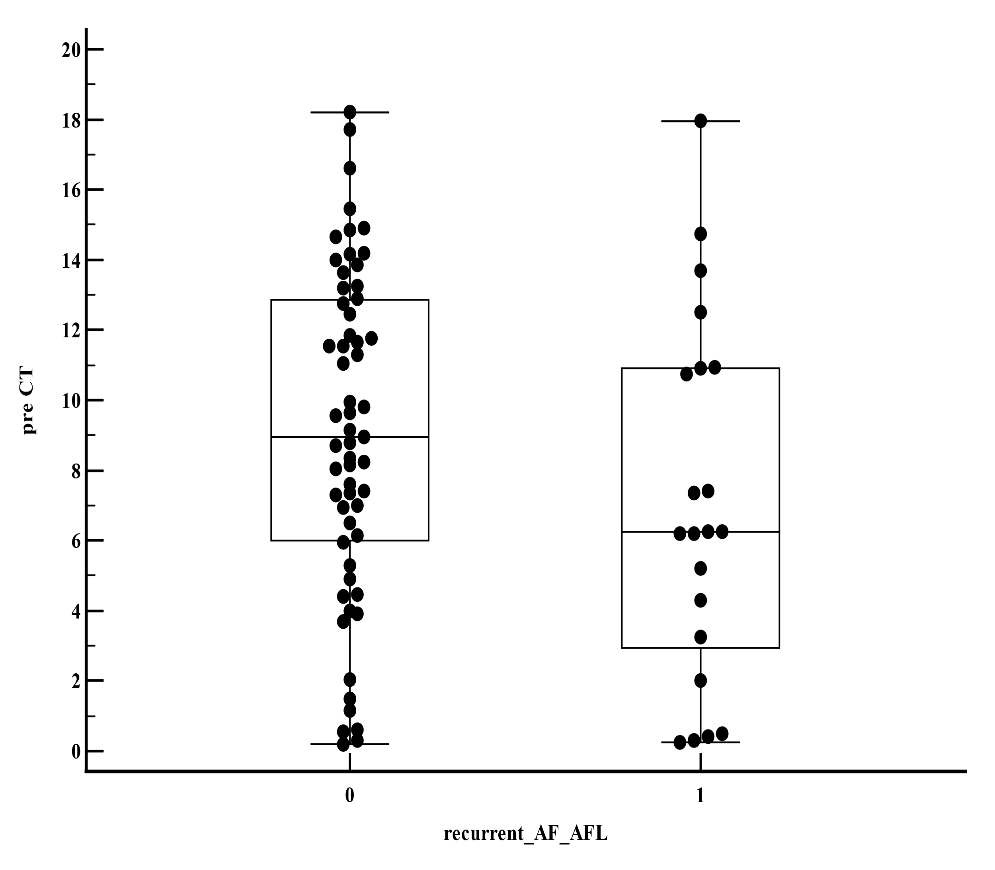

Supplement: Supplementary file 1 — Additional file 1: Multiple comparison graphs of the baseline and post-ablation reservoir (top) and contractile (bottom) strain data in both groups. [file 12968_2021_831_MOESM1_ESM.docx]
